# Bicycling and Walking are Associated with Different Cortical Oscillatory Dynamics

**DOI:** 10.3389/fnhum.2016.00061

**Published:** 2016-02-19

**Authors:** Lena Storzer, Markus Butz, Jan Hirschmann, Omid Abbasi, Maciej Gratkowski, Dietmar Saupe, Alfons Schnitzler, Sarang S. Dalal

**Affiliations:** ^1^Institute of Clinical Neuroscience and Medical Psychology, Medical Faculty, Heinrich Heine University DüsseldorfDüsseldorf, Germany; ^2^Department of Medical Engineering, Ruhr-University BochumBochum, Germany; ^3^Department of Computer and Information Science, University of KonstanzKonstanz, Germany; ^4^Zukunftskolleg and Department of Psychology, University of KonstanzKonstanz, Germany

**Keywords:** EEG, bicycling, walking, motor control, oscillations, sensorimotor cortex

## Abstract

Although bicycling and walking involve similar complex coordinated movements, surprisingly Parkinson’s patients with freezing of gait typically remain able to bicycle despite severe difficulties in walking. This observation suggests functional differences in the motor networks subserving bicycling and walking. However, a direct comparison of brain activity related to bicycling and walking has never been performed, neither in healthy participants nor in patients. Such a comparison could potentially help elucidating the cortical involvement in motor control and the mechanisms through which bicycling ability may be preserved in patients with freezing of gait. The aim of this study was to contrast the cortical oscillatory dynamics involved in bicycling and walking in healthy participants. To this end, EEG and EMG data of 14 healthy participants were analyzed, who cycled on a stationary bicycle at a slow cadence of 40 revolutions per minute (rpm) and walked at 40 strides per minute (spm), respectively. Relative to walking, bicycling was associated with a stronger power decrease in the high *beta* band (23–35 Hz) during movement initiation and execution, followed by a stronger *beta* power increase after movement termination. Walking, on the other hand, was characterized by a stronger and persisting *alpha* power (8–12 Hz) decrease. Both bicycling and walking exhibited movement cycle-dependent power modulation in the 24–40 Hz range that was correlated with EMG activity. This modulation was significantly stronger in walking. The present findings reveal differential cortical oscillatory dynamics in motor control for two types of complex coordinated motor behavior, i.e., bicycling and walking. Bicycling was associated with a stronger sustained cortical activation as indicated by the stronger high *beta* power decrease during movement execution and less cortical motor control within the movement cycle. We speculate this to be due to the more continuous nature of bicycling demanding less phase-dependent sensory processing and motor planning, as opposed to walking.

## Introduction

Walking abilities are severely impaired in patients suffering from freezing of gait, a common phenomenon in advanced Parkinson’s disease (for a review, see Nutt et al., 2011). Surprisingly, bicycling abilities are apparently preserved in the very same patients (Snijders et al., 2011b, 2012). Moreover, bicycling has been put forward as a therapy for Parkinson’s disease, improving motor control, cognitive performance, tremor, and bradykinesia (Ridgel et al., 2009, 2011, 2012). Consequently, contrasting cortical activation during bicycling and walking starting in healthy participants may offer valuable insights into extent and aspects of cortical involvement in motor control of these two distinct types of movement. In addition, it may help to understand why Parkinsonian patients with severe freezing of gait retain the ability to bicycle and it may lead to improved therapeutic applications of bicycling in this patient population.

Vertical ergometer bicycling and walking are characterized by the alternating extension and flexion of the lower limbs that are suggested to be controlled by a shared subset of co-exited muscles (Raasch and Zajac, 1999). Moreover, both are complex movements that recruit a network of motor associated brain regions. Using near-infrared spectroscopy (NIRS; Miyai et al., 2001) and single-photon emission computed tomography (SPECT; Fukuyama et al., 1997), walking was associated with activity in sensorimotor regions, the supplementary motor area (SMA), and cerebellum. Activity within these movement-related brain areas was also reported during recumbent bicycling using functional magnetic resonance imaging (fMRI; Mehta et al., 2009) and positron emission tomography (PET; Christensen et al., 2000). Interestingly, imagination of bicycling compared to rest led to activation in the SMA, while active bicycling compared to passive bicycling was accompanied by a stronger recruitment of the primary motor cortex (Christensen et al., 2000). Furthermore, brain activity was shown to be influenced by exercise intensity and preference (Christensen et al., 2000; Brümmer et al., 2011a,b). This illustrates that the specific functional involvement of movement-related brain areas varies substantially with the task. In this sense, we can expect bicycling and walking to induce different cortical activities as both movements differ, e.g., in the requirement of postural control and coordination. In line with this, a behavioral study by Yogev-Seligmann et al. (2013) indicated that walking depends more on cognitive resources than bicycling. This was indicated by higher interference caused by the additional demand of a secondary task. It remains an open question, however, how exactly walking and bicycling differ on the electrophysiological level.

Previous work in this field using EEG has mainly focused on investigating neural oscillations underlying walking movements and resulted in two main findings.

First, *alpha* (8–12 Hz) and *beta* band (13–30 Hz) activity over the sensorimotor cortex are decreased during active motor execution compared to rest or passive movement (Wieser et al., 2010; Presacco et al., 2011; Severens et al., 2012; Wagner et al., 2012, 2014; Seeber et al., 2014). This tallies with previous findings of power decrease during motor execution, preparation and even during passive and imagined movements (Stančák and Pfurtscheller, 1996; Alegre et al., 2002; Müller-Putz et al., 2007). The decrease is followed by a *beta* power increase (rebound) after movement termination (Pfurtscheller et al., 1996; Parkes et al., 2006; Solis-Escalante et al., 2012). In this sense, *beta* power is modulated dependent on a change of the movement state, i.e., it decreases during the switch from rest to movement and rebounds after the reverse switch from movement to rest.

Second, power modulations are locked to the phase of the gait cycle in the 24–40 Hz and 70–90 Hz range, respectively (Wagner et al., 2012, 2014; Seeber et al., 2014, 2015). Gwin et al. (2011) were the first to show that power is modulated in a cyclic fashion during the gait cycle. Notably, Petersen et al. (2012) found activity at the Cz electrode and the TA muscle to be coherent in the 24–40 Hz range before the heel strike during walking. Although sustained *beta* decrease and transient power modulation in the 24–40 Hz range overlap in the frequency domain, they are thought to be two separate phenomena (Seeber et al., 2014). While sustained *beta* decrease is linked to a sustained active state of the sensorimotor cortex (Seeber et al., 2014; for a review, see Neuper and Pfurtscheller, 2001), phase-dependent power modulation in the 24–40 Hz range is associated with phasic gait cycle-dependent sensorimotor processing and motor planning (Wagner et al., 2012, 2014; Seeber et al., 2014).

Notably, cortical involvement in motor control has also been studied with EEG during recumbent bicycling on a stationary bicycle (Jain et al., 2013). This approach revealed similar findings to the results obtained in the context of walking studies. Active lower leg movements as opposed to passive movements led to a stronger power decrease in the *beta* band over the sensorimotor cortex. Additionally, bicycling was associated with alternating positive and negative cortical potentials, occurring twice in the pedal cycle and correlated with EMG activity of the leg muscles.

In the present study, we contrasted cortical activity underlying bicycling and walking to address the extent and aspects of cortical involvement in motor control. Furthermore, we aimed to clarify which aspects differ across these two distinct types of movement. We investigated movement state-dependent EEG power changes, i.e., the switch from rest to movement and back again. Moreover, we studied movement phase-dependent EEG power changes, i.e., power modulations that are locked to specific phases within the movement cycle.

## Materials and Methods

### Participants

Fifteen healthy right-handed volunteers participated in this study. One participant was excluded from the analyses because of severe contamination of the EEG by muscle artifacts. Thus, data are presented from the remaining 14 participants (24.9 ± 3.0 years, six females). The study was approved by the local ethics committee of the Medical Faculty of the Heinrich Heine University Düsseldorf (study number: 4294) and was performed in accordance with the Declaration of Helsinki. All participants gave their prior written informed consent.

### Experimental Design and Procedure

**Figure 1:**
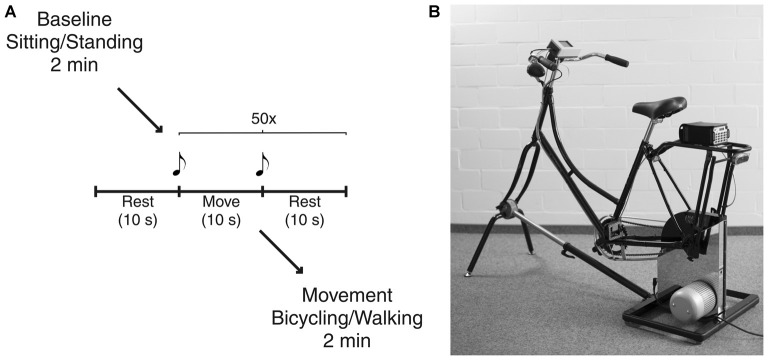
**Experimental setup. (A)** Schematic illustration of the experimental paradigm. Each condition started with a baseline rest period of 2 min, i.e., sitting on the bicycle or standing, respectively. After further 10 s of rest participants performed an alternating sequence containing 10 s of movement, i.e., bicycling or walking, and 10 s of rest. This was repeated 50 times. Start and end of the movement phase were indicated by an acoustic signal. Finally, participants engaged in continuous movement for 2 min. **(B)** The bicycle simulator compromises a Dutch-style bicycle frame mounted on an ergometer. Custom-made software electronics and optical sensors tracked pedal position that was recorded simultaneously with all electrophysiological signals.

Participants started either in the bicycling or in the walking condition (Figure 1A). The order of conditions was pseudorandomized and counterbalanced across subjects. Each condition started with a baseline rest period of 2 min, i.e., sitting on the bicycle or standing, respectively. In order to investigate movement initiation and termination, subjects performed an alternating sequence containing 10 s of movement, i.e., bicycling or walking, and 10 s of rest. The sequence was repeated 50 times. Subjects started with one additional rest phase before continuing with the alternating sequence to capture each of the 50 initiations and terminations. Start and end of the movement phase was indicated by an acoustic signal (beep of 500 ms duration with a frequency of 1000 Hz for start and 1500 Hz for end). Participants were allowed to start with their preferred leg but asked to keep this constant across trials and conditions. Finally, participants engaged in continuous movement for 2 min. The definition of number of repetitions and recording times was based on pilot recordings. Thus, we could ensure to acquire a sufficient data quantity for power analyses. Participants were instructed to bicycle or to walk at a comfortable slow cadence of 40 revolutions per minute (rpm) or strides per minute (spm), respectively. Prior to the experiment, they were given a short trial period to get accustomed to the intended cadence.

Bicycling took place on a basic version of the stationary bicycle simulator originally developed to analyze bicycling performance parameters (Dahmen et al., 2011; Figure 1B). This simulator combines a common bicycle frame with a commercial Cyclus 2 ergometer (RBM Elektronik-Automation GmbH, Leipzig, Germany) based on an eddy current brake. The brake force of the ergometer, controlled by custom-made PC-based software, was kept constant over participants. Bicycling with 40 rpm at a pedal force of 30 N resulted in a power of 23 W. Furthermore, custom Arduino-based electronics and optical sensors tracked pedal position that was recorded simultaneously with all electrophysiological signals. The bicycle simulator was the same for all participants with the saddle height adapted to the individual body height, i.e., the fully stretched leg was matching the distance from the saddle to the lowest pedal position. The walking condition was conducted in a 50 m long hallway with no obstacles or additional cues. Turnings made during continuous walking for 2 min were excluded from the analysis.

### Data Acquisition

Data were recorded with the maximum sampling rate of 2048 Hz using a portable EEG amplifier (Porti amplifier, TMSi, Enschede, Netherlands) and controlled by a combination of the open source software packages OpenBCI and Svarog (Durka et al., 2012). EEG was acquired with an 18-electrode cap (TMSi, Enschede, Netherlands) attached according to the international 10–20 system. EEG signals were referenced against an average reference and a water-based ground electrode integrated in a wristband was used. Bipolar surface EMG activity of three leg muscles (TA, *tibialis anterior*; BF, *biceps femoris*; and RF, *rectus femoris*) was recorded bilaterally using the EEG amplifier and disposable Ag/AgCl electrodes (Covidien, Dublin, Ireland). Electrodes were placed 2 cm apart on the belly of each muscle. Electrode placement was instructed by an expert neurologist in line with the SENIAM guidelines (Hermens et al., 1999). EMG and scalp EEG were recorded with actively shielded cables attenuating movement artifacts. The knee angle was continuously tracked by bilateral electronic goniometers. Footswitches placed under both feet monitored stepping (TMSi, Enschede, Netherlands).

### Artifact Rejection

Data were analyzed with the Matlab-based FieldTrip toolbox (Oostenveld et al., 2011) using Matlab R2014a (The Mathworks, Natick, MA, USA). EEG signals were band-pass filtered offline (1–100 Hz). A band-stop filter was applied (49–51 Hz) to remove power line noise. To account for movement and muscle artifacts contaminating the EEG, the following steps were taken: first, data were visually inspected and times including artifacts such as jumps or transient high frequency activity excluded from further analysis. Second, data were decomposed using independent component analysis (ICA) with the extended Infomax algorithm implemented in the FieldTrip toolbox. The Infomax algorithm has been shown to be superior to two other ICA algorithms, SOBI and FastICA (Delorme et al., 2007). Components showing the spatial or temporal signatures of eye blinks were rejected (1.93 ± 0.47 components per participant; range 1–3) and the remaining components projected back to the scalp channels. Finally, data were re-referenced against the common average of nine central electrodes (F3, Fz, F4, C3, Cz, C4, P3, Pz, P4). By restricting the average reference to these electrodes we were able to minimize the influence of artifacts arising from neck muscles.

### Continuous Movement, Initiation, and Termination of Movement

Power changes during continuous movement were expressed as the relative power change in dB to the baseline rest condition, i.e., sitting and standing, respectively. This was assessed by dividing data into 1 s segments with an overlap of 50% and calculating the fast Fourier transform (FFT) for each segment. Subsequently, spectra were averaged over segments.

The goniometer signal was used to determine movement initiation and termination. The relevant events were defined by the following procedure. First, goniometer amplitude range during movement was determined and set to 100%. Next, baseline levels were calculated for each 2 s period before the acoustic signal which indicated start. A threshold was defined at 2% above the respective baseline level. Movement initiation was then defined as crossing of the predefined threshold. An analogous threshold was defined for movement termination based on the period from 3 to 5 s after the acoustic signal which indicated stop. The accuracy of the selected initiations as well as terminations were verified visually afterwards and adapted if necessary (0% of initiations and 11.60% of terminations).

Movement initiation and termination were analyzed by performing time-frequency decomposition with a sliding Hanning window of 1 s duration and a step size of 0.05 s. Again, power changes were expressed as the relative power change in dB to the baseline rest condition. In order to draw conclusions about differences in relative power between bicycling and walking we checked that baselines were not significantly different using the non-parametric cluster-based permutation test implemented in the FieldTrip toolbox (Maris and Oostenveld, 2007).

### Movement Cycle

The movement cycle was defined as the interval between two consecutive maximum knee flexions of the right knee. The maximum knee flexion is an event occurring both in bicycling and in walking. Therefore, this approach facilitates the comparison between the two types of movement. However, this definition deviates from previous walking studies which used the heel strike as the boundary of the gait cycle (Gwin et al., 2011; Wagner et al., 2012; Seeber et al., 2014). The maximum knee flexion occurs between toe off and heel strike. Power modulations locked to the movement cycle based on the knee flexion precede the ones based on the heel strike by approximately 30% of the movement cycle.

To avoid confounds due to acceleration or deceleration, we solely incorporated cycles deriving from the continuous movement condition and from the period between 2 s after movement start and 2 s before movement stop. This resulted in an average of 202.1 ± 22.2 movement cycles (range 160–240). Time-frequency spectra were calculated per movement cycle with a sliding Hanning window. The window had a length of 0.5 s and was moved in steps of 0.01 s. Individual spectra were interpolated to the length of the longest cycle and subsequently averaged. This procedure standardized the same time frame for all subjects and conditions. Power within the movement cycle was normalized by the temporal average of the entire cycle for each frequency.

Rhythmicity of power modulations within the movement cycle was quantified by the gait phase modulation measure introduced by Seeber et al. (2014) and modified by Trenado (2015). This index can take values between 0 and 1 reflecting the similarity of the power modulation within the movement cycle with a sinusoid. The calculation is based on two periods of the sinusoid representing the modulation with every single leg movement within the movement cycle. The frequency and peak value of maximal sinusoidal modulation within the 24–40 Hz range was computed for every subject and condition. The choice of the frequency range of interest was based on prior studies (Wagner et al., 2012; Seeber et al., 2014). Peak values of the modulation measure did not differ between conditions, i.e., power modulation within the movement cycle had the same similarity with a sinusoid in bicycling and walking (Bicycling: *M* = 0.78, *SD* = 0.13, Walking: *M* = 0.83, *SD* = 0.10, paired *t*-test, *p* > 0.05). The resulting frequency was further used for fitting a sine wave to the corresponding power modulation. Again, a two-period pattern of the sine wave was assumed. This allowed comparing both phase and amplitude of rhythmic power modulations between bicycling and walking.

### EMG Analysis

One of the 14 subjects had to be excluded from the EMG analysis for all muscles and a further subject for one muscle (BF) due to poor data quality. EMG data was band-pass filtered (20–450 Hz) and full-wave rectified. These filter cut-off frequencies were chosen in order to maintain the relevant information and avoiding artifact contamination, especially at lower frequencies (van Boxtel, 2001; De Luca et al., 2010). Average EMG activity was obtained by computing the sum per unit of time. As we did not observe a power difference between body sides in any of the muscles (Bicycling: TA_left *M* = 18.18, *SD* = 15.03, TA_right *M* = 13.93, *SD* = 6.03, BF_left *M* = 19.77, *SD* = 8.34, BF_right *M* = 18.42, *SD* = 8.21, RF_left *M* = 23.41, *SD* = 10.97, RF_right *M* = 21.84, *SD* = 9.50, Walking: TA_left *M* = 49.04, *SD* = 15.26, TA_right *M* = 47.52, *SD* = 10.08, BF_left *M* = 30.70, *SD* = 16.32, BF_right *M* = 31.34, *SD* = 13.32, RF_left *M* = 39.09, *SD* = 23.70, RF_right *M* = 34.47, *SD* = 20.55; paired *t*-tests, all *p* > 0.05; for bicycling TA and walking RF Wilcoxon signed-rank test, all *p* > 0.05), we averaged EMG activity over sides.

Muscle activity within the movement cycle was analyzed as described in Jain et al. (2013). EMG activity was smoothed using a 4th order low-pass Butterworth filter with a cut-off at 5 Hz. EMG activity was summed over left and right muscles, yielding the composite EMG. In contrast to Jain et al. (2013), we interpolated EMG cycles in line with the EEG and computed grand average composite EMG as well as EEG on *z*-score transformed EEG and EMG data. *Z*-score transformed data were obtained by subtracting the mean and dividing by the standard deviation in order to account for scaling differences between subjects. Then, the correlation coefficient was determined between EEG power and EMG activity within the movement cycle.

### Statistics

All analyses were focused on the Cz electrode overlying the leg area of both motor cortices and the SMA. We restricted the analysis to the Cz electrode as previous studies in the context of bicycling and walking were able to demonstrate movement-related activity especially in central sensorimotor areas (Wieser et al., 2010; Gwin et al., 2011; Wagner et al., 2012; Jain et al., 2013; Seeber et al., 2014). Changes of spectral power during continuous movement relative to the baseline rest period were evaluated in three different frequency bands: 8–12 Hz (alpha), 13–22 Hz (low beta), and 23–35 Hz (high beta) based on previous studies (López-Azcárate et al., 2010; Singh et al., 2013; Toledo et al., 2014). The frequency of maximal decrease was obtained for each frequency band and average power at the peak frequency (±1 Hz) compared between bicycling and walking using paired-samples *t*-tests implemented in IBM SPSS statistics, version 22 (IBM Deutschland GmbH, Ehningen, Germany). Differences in time-frequency spectra were evaluated by non-parametric cluster-based permutation testing implemented in the FieldTrip toolbox (Maris and Oostenveld, 2007). The calculation of mean and standard deviation of phase values were performed using the circular variants of these operations using the Circular Statistics Toolbox (Berens, 2009). For evaluation of phase differences between power modulations within the movement cycle we applied a non-parametric permutation test based on Watson’s U^2^ statistic (Zar, 1999). All other variables were tested with SPSS. We tested for normality using the Kolmogorov-Smirnov test. Two-sided paired-samples *t*-tests were performed in case of normally distributed data. Otherwise, we used the non-parametric Wilcoxon signed-rank test. For all tests, the significance level was set to 0.05. We corrected for multiple comparisons using the adaptive Bonferroni correction (Holm, 1979).

## Results

### Movement-Related Power Changes

**Figure 2:**
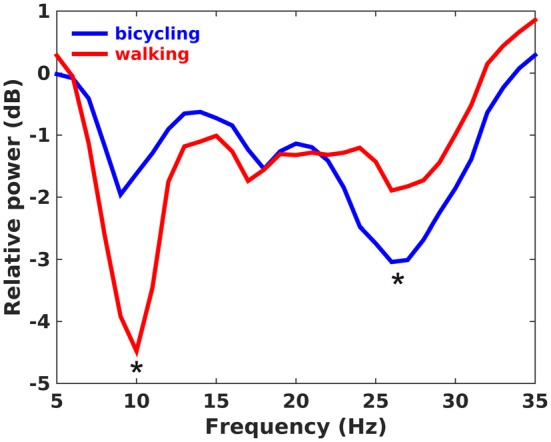
**Power ratio.** Grand average plot showing changes of spectral power in dB during continuous movement relative to the baseline rest period. Positive values indicate a power increase during movement relative to baseline, whereas negative values indicate a power decrease. *Alpha* power decrease was stronger in walking, whereas high *beta* power decrease was stronger in bicycling as indicated by the asterisks.

The average cadence across participants was 40.9 rpm (*SD* = 1.72) for bicycling and 41.5 spm (*SD* = 2.88) for walking. Both bicycling and walking led to a broadband decrease of oscillatory activity relative to the baseline rest condition with minima in the *alpha* (8–12 Hz), low *beta* (13–22 Hz) and high *beta* band (23–35 Hz; Figure 2). For bicycling relative to sitting, *alpha* power decreased about 1.81 dB (*SD* = 1.99) at 10.00 Hz (*SD* = 1.30), low *beta* power about 1.63 dB (*SD* = 1.27) at 18.21 Hz (*SD* = 2.49), and high *beta* power about 3.58 dB (*SD* = 2.27) at 26.71 Hz (*SD* = 2.13). For walking relative to standing, *alpha* power decreased about 4.49 dB (*SD* = 4.06) at 10.14 Hz (*SD* = 1.29), low *beta* power about 1.95 dB (*SD* = 1.98) at 17.71 Hz (*SD* = 1.98), and high *beta* power about 2.30 dB (*SD* = 1.95) at 27.36 Hz (*SD* = 2.84). Frequencies of maximal decrease in the *alpha*, low and high *beta* range didn’t differ between bicycling and walking (all *p* > 0.05), but the pattern of power decreases during movement relative to baseline rest condition was different between the two conditions. While *alpha* power decrease was stronger for walking than for bicycling (*p* = 0.031), the power decrease in the high *beta* band was stronger for bicycling (*p* = 0.046).

**Figure 3:**
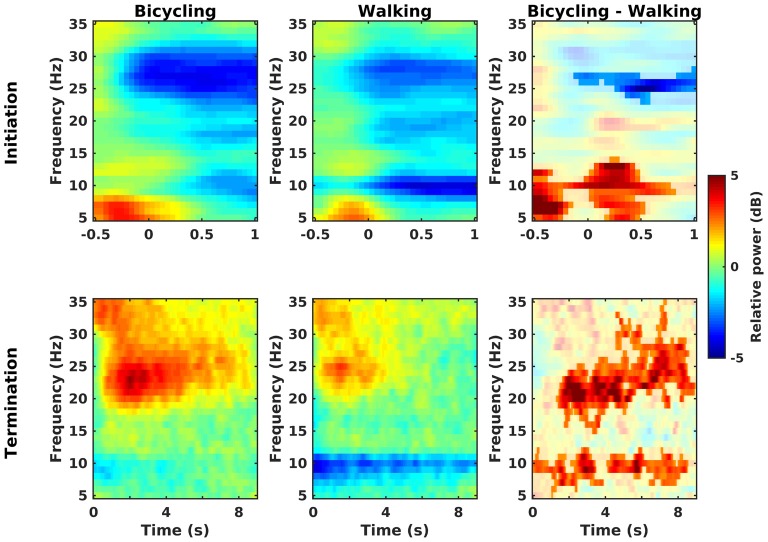
**Initiation and termination of movement.** Grand average time-frequency representations of power changes locked to a change of the movement state relative to the baseline rest period (in dB) for bicycling, walking, and the difference between both (non-significant differences masked). Upper row: Movement initiation is at *t* = 0. In both conditions power decreases (blue) occur in the *alpha*, low and high *beta* band, but with a stronger high *beta* power decrease in bicycling and a stronger *alpha* power decrease in walking. Lower row: Movement termination is at *t* = 0. In both conditions power increase (red) occurs in the *beta* band. Contrasting bicycling and walking revealed a difference in the temporal evolution both of the *beta* rebound and of the *alpha* power recovery.

Power decreases relative to the baseline rest condition could be observed in the *alpha*, low, and high *beta* band at movement initiation in bicycling and walking (Figure 3, upper panel). Furthermore, *theta* power (4–7 Hz) increased both in bicycling and walking relative to the baseline rest condition before movement initiation. For the walking condition, this was followed by a prompt *alpha* power decrease immediately after movement initiation, whereas *alpha* power decreased approximately 500 ms after movement initiation in the bicycling condition. Contrasting relative power between bicycling and walking revealed two significant clusters (Figure 3, upper right panel). One cluster was found in the high *beta* band starting approximately 110 ms before movement start and indicating a stronger power decrease in bicycling compared to walking (*p* = 0.044). Additionally, we observed a significant cluster in the *theta-alpha* range (5–14 Hz, *p* = 0.003) during movement initiation, reflecting a stronger *theta* power increase and weaker subsequent *alpha* power decrease in bicycling compared to walking.

Post-movement *beta* power increase relative to the baseline rest condition could be observed in both conditions about 500 ms after movement termination covering the low and high *beta* range (Figure 3, lower panel). Furthermore, relative *alpha* power recovered immediately after movement stop in bicycling whereas it recovered only gradually after walking. Contrasting relative power between bicycling and walking (Figure 3, lower right panel) showed the post-movement *beta* power increase to be more pronounced and prolonged in bicycling compared to walking (*p* < 0.001). Furthermore, we observed two significant clusters in the *alpha* range (earlier cluster around 0–3.5 s, *p* = 0.036; later cluster around 4–8.5 s, *p* = 0.042) reflecting the persistent post-movement *alpha* power decrease in walking.

### Movement-Cycle Locked Modulations

**Figure 4:**
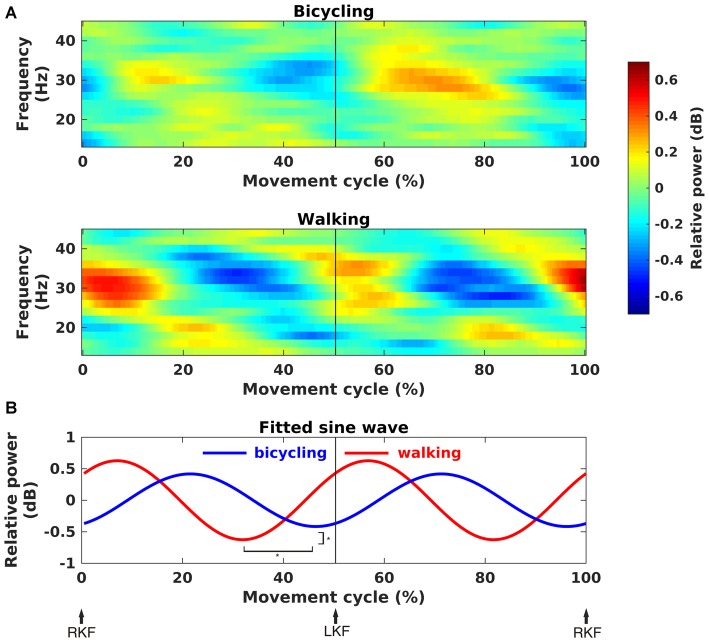
**Movement phase-dependent power modulations. (A)** Grand average time-frequency representations of power changes within the movement cycle relative to the movement cycle mean power (in dB). Sinusoidal power modulations can be observed in the frequency range 24–40 Hz in bicycling (upper panel) and walking (lower panel). **(B)** Grand average sine wave fitted to the power modulation within the movement cycle. The fit was performed for the frequency of maximal movement phase modulation in each individual. It was based on a two-period pattern representing the power modulation with every single leg movement, i.e., right knee flexion (RKF) to left knee flexion (LKF) and LKF to RKF. Sine waves differed significantly in phase and amplitude as indicated by the asterisks.

Time-frequency spectra clearly showed movement cycle-dependent power modulation in the 24–40 Hz range for both bicycling and walking, but with a phase lag between conditions (Figure 4A). There was a significant difference in amplitude (Bicycling: *M* = 0.42, *SD* = 0.17, Walking: *M* = 0.63, *SD* = 0.36, *p* = 0.026) and phase (Bicycling: *M* = −1.09 rad, *SD* = 0.74, Walking: *M* = 1.55 rad, *SD* = 1.06, *p* < 0.001) of the sine wave fitted to the strongest power modulation within the movement cycle (Figure 4B). Power decreases occurred at 29.80% (*SD* = 13.79) and 79.10% (*SD* = 9.71) of the gait cycle when the leading foot was touching the ground and the trailing foot was pushing off. Within the pedal cycle, power decreases occurred shortly before top and bottom pedal positions at 48.87% (*SD* = 12.92) and 92.12% (*SD* = 8.44) of the pedal cycle.

**Figure 5:**
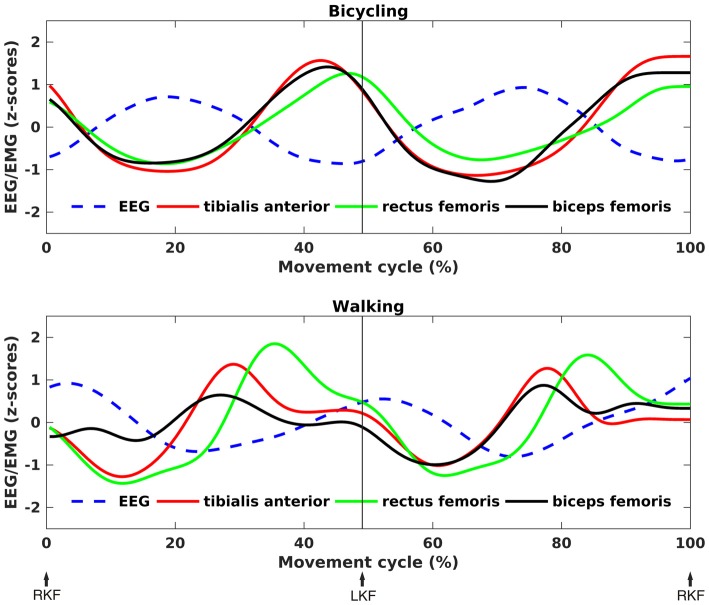
**Correlation between EEG power and EMG activity.** Grand average *z*-score transformed, time-resolved EEG power and EMG activity for bicycling (upper panel) and walking (lower panel). The frequency was subject-specific, i.e., the frequency of maximal movement phase modulation in each individual. An anti-phasic relationship between EEG and EMG can be seen, with EMG activity peaks coinciding with EEG power troughs.

As can been seen in Figure 5, the time points of EEG power decreases coincided with the maximum EMG activity for both conditions. Testing a temporal relationship between the grand average composite bilateral EMG and EEG revealed a negative correlation for all muscles in the bicycling condition (TA: *r* = −0.92, *p* < 0.001; RF: *r* = −0.95, *p* < 0.001; BF: *r* = −0.89, *p* < 0.001). For the walking condition, the TA (*r* = −0.45, *p* < 0.001) and the BF (*r* = −0.44, *p* < 0.001) were correlated with the cortical power envelope. Walking relative to bicycling was accompanied by a stronger average EMG activity of the TA (Bicycling: *M* = 16.06, *SD* = 9.82, Walking: *M* = 48.28, *SD* = 11.40, *p* = 0.006) and the BF (Bicycling: *M* = 19.10, *SD* = 7.76, Walking: *M* = 31.02, *SD* = 12.43, *p* = 0.012). No difference was observed for the average EMG activity of the RF (Bicycling: *M* = 22.62, *SD* = 9.91, Walking: *M* = 36.78, *SD* = 19.46, *p* > 0.05).

## Discussion

Our data demonstrate different cortical oscillatory dynamics subserving bicycling and walking, giving rise to movement specific aspects of cortical involvement in motor control. Bicycling was marked by movement state-dependent power modulations in the *beta* range (13–35 Hz), whereas walking exhibited stronger movement phase-dependent power modulations in the 24–40 Hz range.

### Stronger Movement State-Dependent *Beta* Power Modulation in Bicycling

*Beta* decrease during movement execution as well as *beta* rebound at movement termination was stronger in bicycling than in walking. This is particularly interesting as *beta* power decrease is related to an active state of the sensorimotor cortex associated with increased cortical excitability (Seeber et al., 2014; for a review, see Neuper and Pfurtscheller, 2001), and *beta* rebound to the shift from an active to an idling or even inhibited state of the motor cortex (Pfurtscheller et al., 1996; Solis-Escalante et al., 2012). Overall, *beta* activity is believed to represent maintenance of the current motor state (Engel and Fries, 2010). In this sense, the oscillatory profile of bicycling seems to be dominated by the switch from a resting state to a dynamic state and *vice versa*.

What are the potential reasons for the difference in *beta* power between bicycling and walking? During bicycling, pedals are locked to each other and hence, both legs are moving continuously. In contrast, in walking, each leg is independent with alternating stance and swing phases; i.e., the movement is divided into short and distinct independent entities. In fact, *beta* power was shown to remain suppressed during continuous movements (Erbil and Ungan, 2007; Gwin and Ferris, 2012), whereas it recovered during isometric and sustained movements (Cassim et al., 2000; Gwin and Ferris, 2012). The more continuous motor output in bicycling may therefore result in the stronger *beta* power decrease during movement execution and in a stronger inhibition process at movement termination. Moreover, differences in sensory feedback accompanying motor output might also affect movement state-dependent *beta* power modulation in bicycling (Cassim et al., 2001; van Ede and Maris, 2013).

Alternatively, differences in movement kinematics could have accounted for the difference in *beta* power decrease. Cadence was kept constant across conditions. Still, we were not able to control for differences in velocity, as walking velocity can change at constant cadence by adapting step length. However, central *beta* power decrease was found not to be affected by movement velocity (Stančák and Pfurtscheller, 1996; Bulea et al., 2015). Investigations on the effect of muscular effort on brain activity yielded inconsistent results (Stančák et al., 1997; Christensen et al., 2000; Gwin and Ferris, 2012; Pistohl et al., 2012). In the present study, muscular effort of the TA and the BF were stronger in walking while *beta* decrease was stronger in bicycling. Accordingly, it is quite unlikely that the stronger *beta* decrease in bicycling resulted from stronger muscular effort.

### *Alpha* Power Modulations

Bicycling and walking were associated with different oscillatory dynamics in the *alpha* band. Walking, as opposed to bicycling, was associated with a stronger *alpha* power decrease. Furthermore, *alpha* decrease occurred earlier during initiation of walking, but it was also found to recover later after movement termination. In line with this, Leocani et al. (1997) investigated self-paced finger movements and found cortical areas showing fast *alpha* suppression to be the ones showing late recovery. *Alpha* decrease was shown to persist after movement termination and localized to the postcentral gyrus (Salmelin and Hari, 1994; Salmelin et al., 1995; Leocani et al., 1997; Jurkiewicz et al., 2006). Consequently, *alpha* decrease is assumed to be closely linked to somatosensory processing. This tallies with the findings by Wagner et al. (2012) who showed *alpha* power to decrease during active compared to passive walking in the sensory cortex, probably related to the increased sensory feedback from the muscles. Interestingly, *alpha* power was also reported to decrease in parietal areas during walking with interactive movement related feedback compared to movement unrelated feedback (Wagner et al., 2014). This difference in *alpha* power decrease was suggested to be related to sensorimotor integration during visually guided walking in a virtual environment. Moreover, Presacco et al. (2011) found *alpha* power to decrease during precision walking, i.e., when participants were required to control their foot placement based on visual feedback. As *alpha* band activity is generally associated with attention and information processing (for a review, see Klimesch, 2012), this may point to enhanced sensory and attentional demands in walking.

### Stronger Movement Phase-Dependent Power Modulation in the 24–40 Hz Range in Walking

We report movement phase-dependent power modulation in the 24–40 Hz range for the first time in bicycling and overground walking. Within-cycle power modulation in walking agrees with previous results and has been related to motor planning and sensorimotor processing (Wagner et al., 2012, 2014; Seeber et al., 2014, 2015). However, the exact role remained elusive.

Here, we provide further evidence for cortical oscillatory activity being involved in motor control. For both movement types, power decreases occurred during movement transition phases, i.e., transition between stance and swing phase in walking and flexion and extension in bicycling. Since transition phases occur at different time points in the movement cycle in bicycling and walking if defined according to the maximum right knee flexion (RKF), the corresponding power modulation significantly differed between conditions. This adds to previous results demonstrating strongest negative movement-evoked potentials during transition phases in bicycling and walking, emphasizing highest cortical control during changes of movement direction (Wieser et al., 2010; Jain et al., 2013; Knaepen et al., 2015). In line with this idea, negative potentials have been associated with activity in the cingulate and prefrontal cortex and subsequent positive potentials with activity in sensorimotor regions, thought to reflect processing of afferent feedback (Knaepen et al., 2015). Interestingly, Petersen et al. (2012) found corticomuscular coherence in the 24–40 Hz range between Cz and the TA muscle before the heel strike during walking. Importantly, cortical activity was leading muscular activity, as indicated by the negative sign of the imaginary part of coherency. Together, this may indicate power modulations in the 24–40 Hz range to reflect movement phase-dependent planning processes as an aspect of cortical motor control.

Although bicycling and walking both exhibited movement phase-dependent power modulation with power decreases occurring during movement transition phases, power modulation was found to be stronger in walking. This suggests that bicycling and walking differ in cortical involvement in motor control within the movement cycle. Two factors may contribute to this difference. First, the restricted movement pattern in bicycling with symmetrically moving pedals at fixed circumference may demand less motor planning within the pedal cycle. This assumption is strengthened by the finding of higher interference during walking compared to bicycling in a dual task situation in Parkinson’s disease patients (Yogev-Seligmann et al., 2013). The authors reasoned bilateral coordination making movement vulnerable to cognitive load. Indeed, the SMA has been shown to be involved in interlimb coordination and to be affected in Parkinson’s disease (Wu et al., 2010). Hence, the present difference in cortical activity recorded at the Cz electrode might reflect differences in motor planning due to differing coordination demands.

Second, stronger power modulation in walking might result from stronger muscle recruitment or the integration of related sensory feedback. We found EEG power and EMG activity to be closely correlated in the present study with EEG power decreases coinciding with maximal muscle recruitment of the TA and BF. Notably, average EMG activity of the TA and BF was stronger in walking compared to bicycling. This may be directly related to the difference in cortical power modulation between the two movement types. Moreover, afferent input of the leg muscles was shown to affect cortical activation. Williamson et al. (1997) found passive bicycling to lead to less activation of the motor cortex as compared to active bicycling. However, passive bicycling with additional electrical stimulation of the leg muscles increased the cortical activation in the direction of the level during active bicycling. Somatosensory integration was also suggested to lead to phase specific power increases in the 30–50 Hz range in posterior parietal areas during a speed tracking task on a treadmill when participants had to drive the treadmill speed by themselves, as opposed to a predetermined treadmill speed (Bulea et al., 2015). In agreement with this, integration of sensory information into the motor command was proposed to shift the corticomuscular coherence from the range of 15–30 Hz to higher frequencies, i.e., 30–35 Hz, during dynamic movements (Omlor et al., 2007). Importantly, the huge contribution of sensory afferents to sensorimotor activity was also emphasized by almost identical activation patterns in passive and active bicycling and walking (Wagner et al., 2012; Jain et al., 2013). Interestingly, Wagner et al. (2012) observed a trend for stronger power decreases in active compared to passive walking, whereas Jain et al. (2013) found active compared to passive bicycling to lead to an attenuation of the movement-evoked potentials. They assumed this to be due to sensory gating by efferent corticospinal output. Based on this, phasic sensory input may be more inhibited in bicycling because of the continuous movement pattern and stronger corticospinal drive, respectively.

### Methodological Considerations

Artifact contamination is a major issue in EEG studies on movement (Gwin et al., 2011; Castermans et al., 2014; Kline et al., 2015). Recently, several artifact rejection methods, e.g., ICA in combination with dipole analysis, have been applied to study oscillatory activity during walking (Gwin et al., 2011; Wagner et al., 2012). We decided to use ICA solely for rejecting components clearly reflecting eye blinks and not components potentially related to muscle artifacts. Due to the small number of electrodes ICA cannot be expected to isolate muscle artifacts completely, i.e., many of the components will be a mixture of artifacts and brain activity, and should not be discarded. Moreover, we restricted the average reference to the nine central electrodes in order to minimize muscular artifact contamination especially from the neck muscles which mostly affected the lateral and occipital electrodes. However, we cannot rule out that we thereby changed signal localization and power or artifacts remaining in the data. Notably, the similarity between our data and prior results suggests that our conservative procedure yielded comparable data quality. This point is additionally supported by intra-cycle power modulations occurring in a physiologically limited frequency band (24–40 Hz) and not as broadband activity. This would be expected in case of movement artifact contamination (Castermans et al., 2014) and in particular muscular artifact contamination from the neck muscles (Gwin et al., 2011; Severens et al., 2012).

Interpreting the differences in cortical activity underlying bicycling and walking one has to keep in mind that several factors as e.g., exercise intensity and preference influence brain activity (Brümmer et al., 2011a,b). We controlled for a difference in cadence as Christensen et al. (2000) have shown that cortical activation correlates with cadence but not with pedal resistance during recumbent bicycling. However, as we did not measure heart beat or direct calorimetry, we cannot directly control the influence of physiological load on the power difference between bicycling and walking.

Furthermore, participants had to bicycle and walk with a predetermined cadence of 40 rpm and 40 spm, respectively. The imposed cadence might have deviated from the self-preferred cadence which could have influenced brain activity during both movements, especially cortical control within the movement cycle. A relationship between cortical control and walking cadence is suggested by the findings of slowed walking cadence in the elderly under dual-task load (for a review, see Al-Yahya et al., 2011). Not controlling for a difference in cadence, however, would have caused an obvious mismatch in motor output between bicycling and walking. Furthermore, Gwin et al. (2011) did not find any significant difference comparing gait phase-dependent EEG power modulation for 0.8 and 1.25 m/s walking.

### Implications for Freezing of Gait in Parkinson’s Disease

The finding of different cortical oscillatory dynamics in bicycling and walking may shed some light on the puzzling finding of preserved bicycling abilities in Parkinson’s patients suffering from severe freezing of gait (FOG; Snijders et al., 2011b, 2012). FOG has been associated with impaired temporal and spatial gait control (Nieuwboer et al., 2001; Hausdorff et al., 2003), decreased activity of the SMA (Snijders et al., 2011a), and increased resting state high *beta* power within the subthalamic nucleus (Toledo et al., 2014). Parkinson’s disease is generally associated with abnormally increased *beta* band activity in the corticobasal ganglia loop (for a review, see Oswal et al., 2013). Therefore, one may speculate that bicycling may be less hampered in FOG because the more continuous nature of this movement promotes *beta* suppression and demands less sensorimotor processing. Furthermore, the more continuous movement in bicycling may be less vulnerable to interruptions than the independent movement entities in walking. A study specifically addressing this hypothesis is in progress.

## Conclusion

The present study provides insight into oscillatory dynamics being involved in motor control during both bicycling and walking. We found stronger movement state-dependent high *beta* power modulation but less movement phase-dependent power modulation in the 24–40 Hz range in bicycling as opposed to walking. Our data suggest that, relative to walking, bicycling is characterized by a stronger cortical activation. This may be the result of the more continuous movement pattern, precluding a regular rest period. In contrast, phases of rest and movement alternate all the time in walking and may therefore demand more phase-dependent sensory processing and motor planning.

## Author Contributions

All authors contributed to the design of the experiment and revised the manuscript. LS, MB, JH, OA, and SSD were involved in data analysis and interpretation and LS further in data acquisition. All authors approved the final version of the manuscript, agreed to be accountable for all aspects of the work and qualify for authorship.

## Conflict of Interest Statement

The authors declare that the research was conducted in the absence of any commercial or financial relationships that could be construed as a potential conflict of interest.
